# Energy landscape and dynamic state modeling for schizophrenia biomarker extraction in task-based fMRI

**DOI:** 10.1371/journal.pone.0339756

**Published:** 2026-10-08

**Authors:** Janerra D. Allen, Elliot Hong, Fow-Sen Choa

**Affiliations:** 1 Johns Hopkins University Applied Physics Lab, Laurel, Maryland, United States of America; 2 Department of Computer Science and Electrical Engineering, University of Maryland Baltimore County, Baltimore, Maryland, United States of America; 3 Department of Psychiatry and Behavioral Sciences, UT Health, Houston, Texas, United States of America; IPM: Institute for Research in Fundamental Sciences, IRAN, ISLAMIC REPUBLIC OF

## Abstract

Task-based functional magnetic resonance imaging (fMRI) examines how the brain dynamically responds to cognitive and perceptual demands, offering complementary insight beyond traditional activation-based analyses in schizophrenia. Prior task-based fMRI studies have identified reduced functional connectivity within auditory and associated cortical areas. In this study, we investigated task-evoked functional connectivity and brain state dynamics in 25 healthy controls, 23 patients with schizophrenia experiencing auditory verbal hallucinations (AVH+), and 23 patients without hallucinations (AVH-). Participants completed multiple auditory paradigms, including word lists, sentence lists, and reverse speech. The most robust connectivity differences emerged during the word list task, where cluster-level analyses revealed distinct network interaction patterns differentiating controls, AVH + , and AVH-patients. Energy landscape modeling further demonstrated altered stability and organization of brain states in both patient groups, with the largest deviations observed in AVH+ individuals. These alterations point to dysregulated neural dynamics linked to hallucination vulnerability. Overall, the results show that task-based fMRI sensitively captures disruptions in functional connectivity and brain state stability relevant to auditory hallucinations, underscoring its value for characterizing network-level mechanisms underlying perceptual disturbances in schizophrenia.

## Introduction

Functional magnetic resonance imaging (fMRI) has become a cornerstone of human brain mapping, providing critical insights into intrinsic and stimulus-driven neural processes. Resting-state fMRI (rs-fMRI) has revealed consistent patterns of functional connectivity and large-scale brain networks, offering a foundational view of neural organization in both healthy and diseased brains [1,2]. However, resting-state paradigms lack explicit task demands and are limited in capturing dynamic, context-dependent neural responses that underpin cognition and psychiatric symptoms. Moreover, dynamic functional connectivity analyses have revealed that resting-state networks exhibit time-varying patterns, underscoring the need for task-based paradigms to capture transient cognitive states [3]. In contrast, task-based fMRI (tb-fMRI) enables researchers to probe how specific cognitive, perceptual, or motor tasks engage distributed brain networks, resulting in behaviorally anchored neural signatures [4,5].

Schizophrenia, a complex neuropsychiatric disorder marked by disturbances in perception, cognition, and behavior, presents a compelling case for task-based neuroimaging. Although rs-fMRI studies have highlighted widespread dysconnectivity in default mode, sensorimotor, and thalamocortical networks, these findings often provide generalized signatures that lack specificity for individual symptoms or subtypes [6,7]. Task-based approaches help address this gap by capturing how brain networks respond to cognitive load, offering more precise insights into the neural mechanisms underlying core symptoms such as auditory verbal hallucinations (AVH), delusions, and working memory impairments. Recent studies using symptom-capture and neurofeedback paradigms have demonstrated that task-based fMRI can isolate AVH-specific network disruptions, particularly in frontotemporal and cingulate regions [8–10].

Among these, AVH remains one of the most intriguing and debilitating symptoms. Hallucinations reflect a failure in the brain’s ability to distinguish internally generated representations, such as inner speech, from externally driven sensory input. Neuroimaging studies have consistently implicated abnormal activation in the auditory cortex, speech-motor areas, and prefrontal regions during AVH episodes [11]. This supports the view that hallucinations arise from dysfunctions in self-monitoring, sensory integration, and internal model updating. These dysfunctions are consistent with predictive coding frameworks, which posit that hallucinations are the result of an imbalance between top-down priors and bottom-up sensory input [12,13].

Crucially, hallucinations are not isolated errors in perception, but dynamic processes shaped by brain state transitions. They may arise when unstable or aberrant network dynamics leads to spontaneous activations of sensory representations, even in the absence of corresponding stimuli. From a systems-level perspective, these dynamics result from impaired functional connectivity, particularly between control hubs (e.g., prefrontal cortex) and sensory areas—combined with a failure to suppress internally generated predictions. Task-based fMRI is uniquely positioned to reveal these mechanisms by forcing the brain to engage with structured stimuli and observing deviations from normative responses in real time.

To characterize these dynamics, this study applies energy landscape modeling, a framework drawn from statistical physics that visualizes brain activity as traversals through a landscape of stable and unstable states. In this model, brain states correspond to attractors in a high-dimensional space, and transitions reflect shifts in network configurations. In healthy brains, tasks evoke transitions into stable, goal-directed states. In schizophrenia, especially in individuals with AVH, these landscapes may become shallow, noisy, or fragmented, making the brain more susceptible to falling into maladaptive or hallucinatory attractors. Previous applications of energy landscape analysis in schizophrenia have identified fragmented attractor states and reduced network stability, supporting its utility in modeling psychiatric symptoms [14,15].

This study takes advantage of these parallels to investigate hallucination generation from a unified perspective. We analyze task-evoked fMRI data from three groups: healthy controls (CON), schizophrenia patients with AVH (AVH+) and those without AVH (AVH-). Using language-processing tasks known to activate the auditory and prefrontal regions implicated in AVH, we examine how brain activation, functional connectivity, and dynamic transitions differ between groups.

Our approach integrates task-based fMRI with energy landscape modeling to characterize alterations in the stability and variability of functional brain states. By comparing hallucinating and non-hallucinating patients, we aim to identify neural signatures that distinguish pathological internal representation from normative perceptual processing.

This framework provides a novel account of AVH as a failure of context-sensitive generative control. Beyond deepening our understanding of schizophrenia, the results may contribute to a broader theory of hallucination as a failure mode in predictive systems—biological or artificial.

## Materials and methods

### Data characteristics

Twenty-five healthy controls and forty-six individuals diagnosed with schizophrenia were included in this study Table 1. The schizophrenia cohort was further subdivided based on clinical presentation: 23 patients who frequently experienced auditory verbal hallucinations (AVH+) and 23 who did not report hallucinations (AVH-) (Table 2). Data were obtained from the publicly available dataset associated with Soler-Vidal et al. (2022) [16].

**Table 1:** Demographics: Healthy controls vs. patients.

	CON (*N* = 25)	PAT (*N* = 46)	Differences
Age	39.80 ± 14.08	42.52 ± 10.72	t=−0.84,p=0.40
Sex	18 (Male)	36 (Male)	$\chi^{2}=0.35,p=0.55$
	7 (Female)	10 (Female)	
IQ	101.17 ± 9.91	99.82 ± 9.27	*t* = 0.55, *p* = 0.59
PSYRAT-H	n/a	12.46 ± 13.60	n/a

**Table 2:** Demographics: Patient subgroups (AVH- vs. AVH+).

	AVH- (*N* = 23)	AVH+ (*N* = 23)	Differences
Age	44.96 ± 7.38	40.09 ± 12.96	*t* = 1.57, *p* = 0.13
Sex	16 (Male)	20 (Male)	$\chi^{2}=2.04,p=0.15$
	7 (Female)	3 (Female)	
IQ	101.32 ± 8.89	98.39 ± 9.60	*t* = 1.06, *p* = 0.29
PSYRAT-H	0	24.91 ± 7.32	t=−16.32,p<0.001

IQ: intelligence quotient; PSYRATS-H: Psychotic Symptom Rating Scale. Values are means (± SD).

### Data acquisition

The participants were recruited from psychiatric services in Spain, as reported by Soler-Vidal et al. The study received ethical approval from the Research Ethics Committee FIDMAG Sisters Hospitallers. Our analysis used fully de-identified, publicly available data; the authors of this study had no access to information that could identify individual participants. The dataset was accessed for research purposes in approximately May 2024.

The dataset includes functional MRI data collected during a block design consisting of three auditory experimental conditions: word lists, sentence lists, and reverse speech. Clinical measures included estimated IQ and the Psychotic Symptom Rating Scale for Auditory Hallucinations (PSYRATS-H), a validated multidimensional scale assessing AVH features such as frequency, distress, and controllability [17,18].

Although all three tasks were collected and initially analyzed, this study focuses primarily on the word list task. This choice was made *a priori* because the word list paradigm provides the strongest and most consistent engagement of auditory and language networks implicated in AVH, including primary auditory cortex, superior temporal gyrus, and prefrontal monitoring regions. These regions are central to prevailing mechanistic models of auditory hallucinations and to the hypotheses of this study. The focus on this task therefore reflects theoretical relevance and neural specificity, not data-driven selection (secondary exploratory task contrasts are provided in S1 Appendix).

### Data preprocessing and denoising

Preprocessing followed the approach described by Allen et al. [19], using the CONN and SPM toolboxes for functional connectivity analysis, as detailed in the CONN handbook [20,21]. Steps included anatomical CompCor denoising, task regressors, and ROI-to-ROI clustering methods tailored for task-based designs. Adjustments were made to incorporate stimulus-event regressors and to model condition-specific BOLD dynamics. The CONN toolbox was used for both block design preprocessing and structured denoising, isolating task-related neural fluctuations from intrinsic activity. Denoising incorporated anatomical CompCor, a component-based noise correction method that reduces physiological and motion-related artifacts [22]. Complete procedural configurations, including motion scrubbing parameters, filtering bandwidths, and software pipeline scripts, are detailed in S1 Appendix and archived in our public GitHub repository (https://github.com/janerradallen/sz-task-based).

### First-level analysis

At the individual level, functional connectivity was computed using 246 regions of interest (ROIs) from the Brainnetome atlas [23]. To facilitate network-level interpretation, these subregions were mapped onto 9 canonical intrinsic connectivity networks (AUD, ATN, CON, DMN, FPN, SAN, SSM, SUB, VIS) extending the Automated Anatomical Labeling (AAL)-inspired subcortical and cortical network assignments (full MNI coordinates and network assignments are listed in S1 Table and S1 File). The seed-to-voxel and ROI-to-ROI connectivity metrics were calculated using Fisher-transformed bivariate correlation coefficients, modeled via a weighted general linear model (GLM) [21]. Task regressors were convolved with the canonical hemodynamic response function to account for condition-specific BOLD fluctuations. Condition-specific activation and connectivity were estimated using the GLM framework [24], with scan weights adjusted for task timing to capture transient neural responses.

### Second-level (group-level) analysis

Group-level analysis used a GLM to compare task-modulated connectivity across groups. Functional Network Connectivity (FNC) and Spatial Pairwise Connectivity (SPC) were used to define and analyze connection clusters, adapted from the methods used in resting-state studies [25,26]. Connectivity estimates were derived from contrast maps that isolate condition-specific responses to auditory stimuli. Statistical inference was performed using Random Field Theory to control for family-wise error rates [26].

#### Functional network connectivity.

Functional network connectivity (FNC) was used to analyze groups of functionally related ROIs, using hierarchical clustering to define connectivity networks based on anatomical proximity and task-dependent functional similarity. Connectivity matrices were examined for patterns within and between networks, revealing task-modulated connectivity effects. FNC statistics included F-values for each ROI pair, with uncorrected and FDR-corrected p-values to identify significant clusters. Task-related changes in functional connectivity were visualized as connectome rings, highlighting significant ROI-to-ROI correlations [27].

#### Spatial pairwise connectivity.

Spatial pairwise connectivity (SPC) was applied to the ROI-to-ROI connectivity matrices for each task condition, using optimal leaf ordering to refine hierarchical clustering and improve cluster interpretability [28]. Analysis followed the same cluster-wise inference framework as the resting-state literature, with connectivity differences assessed based on task conditions. SPC results included T- or F-statistic maps thresholded to identify significant clusters, with uncorrected and FDR-corrected p-values capturing task-modulated connectivity patterns. Suprathreshold clusters were iteratively refined to estimate the probability density of task-related connectivity effects under the null hypothesis, ensuring robust detection of stimulus-driven functional connectivity changes [27]. Cluster significance was assessed using topological false discovery rate (FDR) correction, which accounts for spatial dependencies in neuroimaging data [27].

In CONN’s SPC clustering inference measure, the terms score, mass, and size describe different properties of connectivity clusters. The score reflects the strength of individual connections and is typically represented as a t-score or z-value. The mass summarizes the total accumulated connectivity strength across the cluster, and the size reflects to the number of connections within the cluster. Thus, score indicates connection strength, size reflects spatial extent, and mass provides an integrated measure of both.

Together, these statistical frameworks—GLM modeling, Random Field Theory, topological FDR, and hierarchical clustering—ensure robust detection of task-driven connectivity differences across clinical groups.

### Statistical testing procedures

To assess task-dependent functional connectivity differences across groups, second-level analyses were performed using general linear models (GLMs). Connectivity estimates were derived from contrast maps isolating condition-specific responses. Two methods were used to identify significant clusters of ROI-to-ROI connectivity: Functional Network Connectivity (FNC) and Spatial Pairwise Connectivity (SPC), both implemented in the CONN toolbox.

Cluster-level inference was performed using F-statistics or T-statistics (depending on the comparison), followed by Bonferroni correction to account for multiple comparisons. For each task condition, the total number of possible clusters was used to adjust the significance threshold (e.g., α = 0.05 / total clusters), controlling for Type I error in the context of multiple testing.

### REX – Neuroimaging toolbox and energy landscape modeling

To prepare inputs for energy landscape modeling, regions of interest (ROIs) corresponding to Bonferroni-corrected significant clusters were extracted from the connectivity analyses. Task-based fMRI time series were obtained for each subject by spatially aligning the 246-region Brainnetome atlas [23] to denoised, post-processed functional images using REX, a MATLAB-based toolbox for ROI analysis [29]. This procedure yielded a matrix of dimensions 341 × 246, representing time points and ROI-specific BOLD signals. For subsequent analyses, we focused on a subset of ROIs identified through SPC-based clustering, which showed the strongest differences between groups following Bonferroni correction. The original matrix was transposed and subset by ROI and task condition to generate reduced time series matrices for each comparison group.

These reduced matrices served as input to a pairwise Maximum Entropy Model (MEM) to construct the brain’s energy landscape. Here, “statistical energy” *E*(*S*) is strictly defined as a logarithmic metric of network state probability ($P(S)\propto e^{-E(S)}$), where lower energy values denote higher occurrence probabilities (basins of attraction) within the network state space. This formulation characterizes statistical state stability and does not reflect biological or metabolic energy consumption (see S1 Appendix for detailed mathematical derivations, binarization protocols, and model fitting algorithms).

### Energy landscape analysis

The energy landscape (EL) analysis framework was used to characterize state-space dynamics across the reduced sets of ROIs identified via Bonferroni-corrected clustering. Energy landscape analysis characterizes the permissible functional states of a network, illustrating how binary activity configurations evolve over time. This approach is grounded in the pairwise maximum entropy model (MEM), which fits second-order statistical models to empirical data to capture dynamic fluctuations in network activity [14,15,30–33].

At each ROI (i=1,...,N) and time point *t*, the BOLD signal was binarized into active (+1) or inac*t*ive (−1) states by thresholding relative to the ROI temporal mean. The empirical frequency of each binary activity pattern was then computed. For *N* ROIs, there are $2^{N}$ possible brain states. Using MEM, energy values *E*(*S*) and probability occurrences *P*(*S*) were estimated for each state. In this statistical physics formulation, energy and probability are inversely related ($P(S)\propto e^{-E(S)}$): states with lower statistical energy occur more frequently and correspond to deeper attractor basins in network state space. We emphasize that statistical energy represents state probability density rather than metabolic or biological energy expenditure. The pairwise MEM is preferred for its balance between computational tractability and its ability to capture essential pairwise interactions without overfitting.

Conceptually, the energy landscape provides a topological framework for visualizing the stability of and transitions between brain states, where low-energy basins correspond to statistically stable functional configurations (attractors), and transitions between basins reflect dynamic reconfigurations in connectivity. While EL modeling has been extensively validated in resting-state fMRI [31,32], its application to task-based data allows for characterization of stimulus-driven dynamic state transitions [15,30].

In the present analysis, primary EL modeling was focused on the word-list task to test our *a priori* hypotheses regarding language and auditory network dysregulation in AVH. To ensure dimensional tractability and interpretability, EL modeling was applied to reduced ROI subsets derived from both Functional Network Connectivity (FNC) and Spatial Pairwise Connectivity (SPC) analyses. While downstream state-space modeling was primarily executed on the word-list task, exploratory comparisons across secondary tasks (sentence list, reverse speech, and white noise) were also conducted and are detailed in S1 Appendix.

### Paired states and statistical comparison

Each subject exhibited a distribution across $2^{N}$ possible connectivity states, each associated with a specific statistical energy value. Energy values for all states were computed across participants. Two-sample *t*-tests were conducted *t*o compare state energy profiles between control and AVH+ groups, as well as between AVH+ and AVH- groups. Bonferroni correction was applied with a significance threshold of α=0.05, adjusted for the total state-space size ($2^{N}$).

Complementary paired states (configurations with inverted activation vectors) were identified within each subgroup based on their energy profiles to evaluate symmetry in functional brain dynamics across clinical cohorts.

### Energy-based state dynamics

Task-based fMRI captures neural activity in response to cognitive demands, enabling the study of network-level state trajectories through high-dimensional energy landscapes [14,15,30–33]. Low-energy states correspond to frequently visited, statistically stable attractor configurations, whereas high-energy states represent transient, less frequently occupied transitions.

In schizophrenia, particularly in patients with auditory verbal hallucinations (AVH+), deficits in sensory gating, attention, and predictive monitoring [3,5] may manifest as altered energy landscape topography—such as abnormal basin depth or altered state stability. We hypothesized that AVH+ patients would exhibit altered energy landscapes relative to controls and AVH- patients during the word-list task, characterized by altered occupancy rates in auditory-DMN co-activation states. Interpretations of energy-based state dynamics as indices of cognitive stability or flexibility remain exploratory and theoretically grounded [14,15,30].

### State transition dynamics

Analyzing transitions between brain states provides insight into the dynamic reconfiguration of functional networks. Dwell time—defined as the continuous duration the network remains within a given basin of attraction—is a key metric in energy landscape analyses [3,14,31–33]. Shorter dwell times indicate rapid state shifts and network instability, whereas prolonged dwell times in specific basins suggest trapping in local minima.

In AVH+ schizophrenia, altered state dynamics may reflect prolonged occupancy in hyperconnected or maladaptive prior-driven states at the expense of flexible, task-directed configurations [15,30]. Energy-based metrics—including visit counts, dwell times, and transition probability matrices—quantify these temporal dynamics. We hypothesized that AVH+ individuals would exhibit distinct dwell-time distributions and altered transition probabilities relative to AVH- and control groups during auditory task performance.

## Results

### Group-level connectivity overview

We first examined ROI-to-ROI functional connectivity across all auditory tasks. Individual connectivity comparisons revealed no significant differences between controls and all patients, controls and AVH- patients, controls and AVH + , or between male and female participants.

At the level of individual connections, no pairwise connectivity differences survived strict correction for multiple comparisons across groups (CON, AVH-, AVH + , male, female), consistent with previous observations that schizophrenia-related dysconnectivity is often broadly distributed rather than localized [1,3]. However, when considering average connectivity across clusters, significant differences emerged between controls and AVH+ participants, as well as between AVH- and AVH+ participants. This pattern indicates that network-level reconfigurations, rather than isolated edge disruptions, underlie task-related dysconnectivity in schizophrenia.

Functional connectivity analyses were conducted across all auditory conditions (word lists, sentence lists, reverse speech, and white noise) using both Functional Network Connectivity (FNC) and Spatial Pairwise Connectivity (SPC) clustering. While functional mapping was completed across all conditions, the *a priori* prioritized word-list task yielded the most robust and statistically significant group differences. Accordingly, main text findings focus on the word-list task, while comprehensive results for secondary exploratory tasks (sentence list, reverse speech, and white noise) are detailed in S1 Appendix.

**Control vs. All Patients.** Comparing healthy controls (*N* = 25) to all patients with schizophrenia (*N* = 46), no significant clusters were observed in either FNC or SPC analyses across any task after Bonferroni correction.

**Control vs. AVH+ Patients.** During the word-list task, significant differences were detected. FNC identified 3 significant clusters comprising 11 ROIs, and SPC identified 1 cluster comprising 4 ROIs (see Tables 3 and 4). For secondary tasks, FNC and SPC identified clusters that did not survive Bonferroni correction, except in specific sub-contrasts (detailed in SI Section S3).

**Table 3:** Statistical cluster analysis for the between-subject [Control (−1) vs. AVH+ (+1)] contrast using Functional Network Connectivity (FNC) for the *word-list* task (post-Bonferroni correction).

Analysis		Statistic	*p*-unc	*p*-FDR
Cluster 1		F(2,45) = 15.14	< 0.001	0.010
Left lPFtha	Right mPFtha	T(46) = 5.55	< 0.001	0.001
Right lPFtha	Right mPFtha	T(46) = 5.32	< 0.001	0.001
Right mPMtha	Right lPFtha	T(46) = 4.64	< 0.001	0.010
Cluster 2		F(2,45) = 10.64	< 0.001	0.050
Left PPtha	Left V5/MT+	T(46) = −4.77	< 0.001	0.001
Left PPtha	Right V5/MT+	T(46) = −4.65	< 0.001	0.001
Cluster 3		F(2,45) = 10.57	< 0.001	0.050
Right A4hf	Right A4t	T(46) = 4.62	< 0.001	0.001
Left A4hf	Left A4t	T(46) = 4.49	< 0.001	0.010

*p*-unc: uncorrected *p*-value; *p*-FDR: false discovery rate-adjusted *p*-value.

**Table 4:** Statistical cluster analysis for the between-subject [CON (−1) vs. AVH+ (+1)] contrast using Spatial Pairwise Connectivity (SPC) for the *word-list* task (post-Bonferroni correction).

Analysis		Statistic	*p*-unc	*p*-FDR
Cluster 1		Score = 113.86	< 0.001	0.030
		Mass = 161.35	< 0.001	0.010
		Size = 6	< 0.001	0.030
Left lPFtha	Right mPFtha	T(46) = 5.55	< 0.001	0.030
Right lPFtha	Right mPFtha	T(46) = 5.32	< 0.001	0.030
Right mPMtha	Right lPFtha	T(46) = 4.64	< 0.001	0.090

*p*-unc: uncorrected *p*-value; *p*-FDR: false discovery rate-adjusted *p*-value.

**AVH + vs. AVH- Patients.** In this subgroup comparison (*N* = 23 per group), SPC identified significant clusters during the word-list task after Bonferroni correction. Additional exploratory contrasts for sentence and reverse speech conditions are compiled in S1 Appendix.

Overall, these findings confirm that the word-list task captured the most consistent group differentiation involving AVH+ participants. Subsequent Maximum Entropy Model (MEM) energy landscape analyses were therefore focused on this task.

### Functional network connectivity

#### Connectivity maps: AVH + vs. CON – Word list task.

To investigate global connectivity patterns during the word-list task, we examined functional connectivity between CON and AVH+ groups. The unthresholded connectome ring demonstrates extensive correlations across all regions of interest (ROIs) prior to adjustment for multiple comparisons (Fig 1). After applying Bonferroni correction, the connectivity map was refined to display only significant functional network connectivity (FNC) signatures (Fig 2). Nodes in these displays are grouped by functional network affiliation, with weak edges (*r* < 0.30) filtered to highlight principal network drivers (SI Section S5).

**Fig 1:**
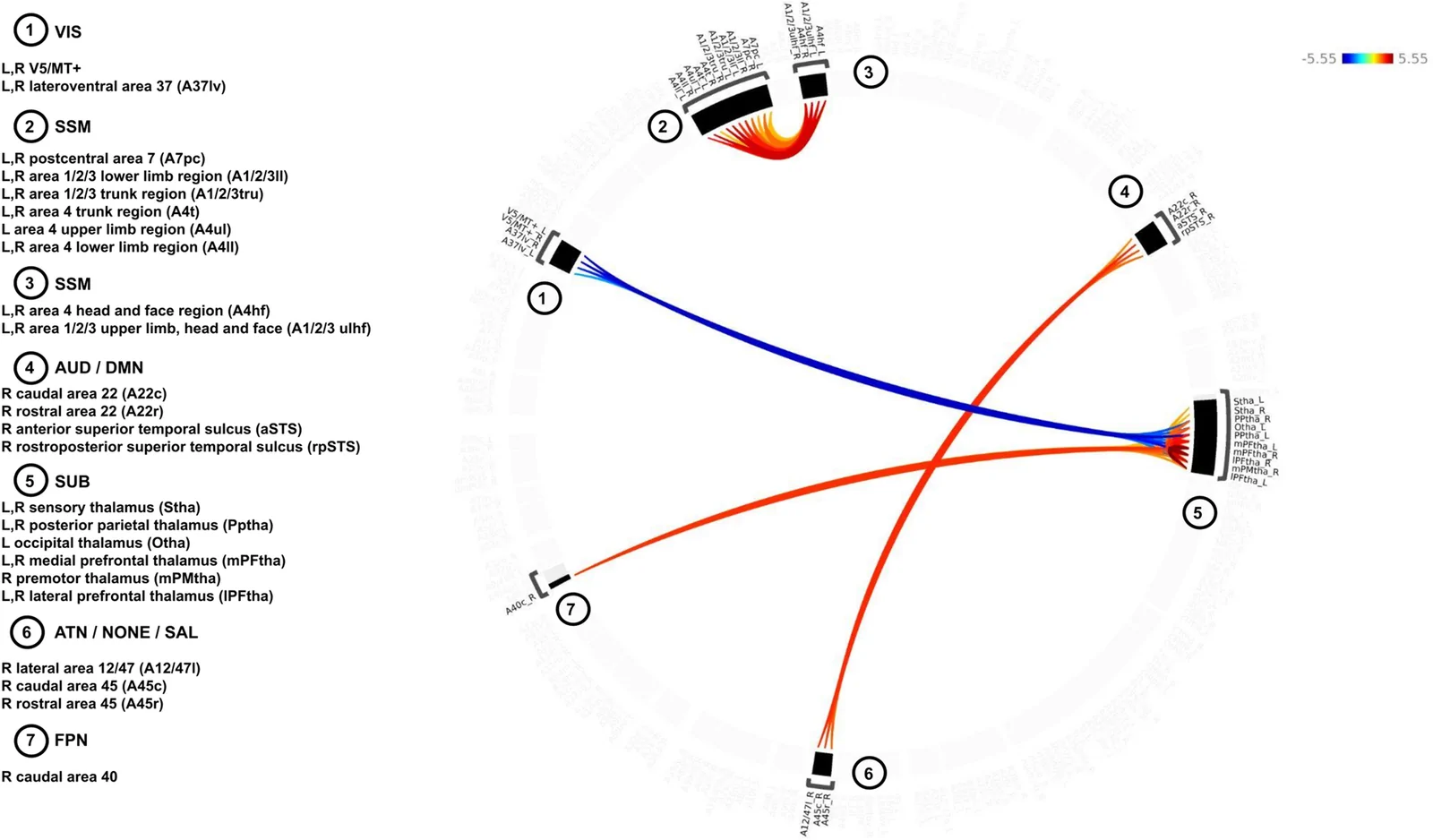
Mean functional connectivity for the between-subject [CON (−1) vs. AVH+ (+1)] contrast presented as an unthresholded connectome ring for the *word-list* task (before Bonferroni correction) computed using Functional Network Connectivity (FNC). CON: healthy controls; AVH + : patients with auditory verbal hallucinations.

**Fig 2:**
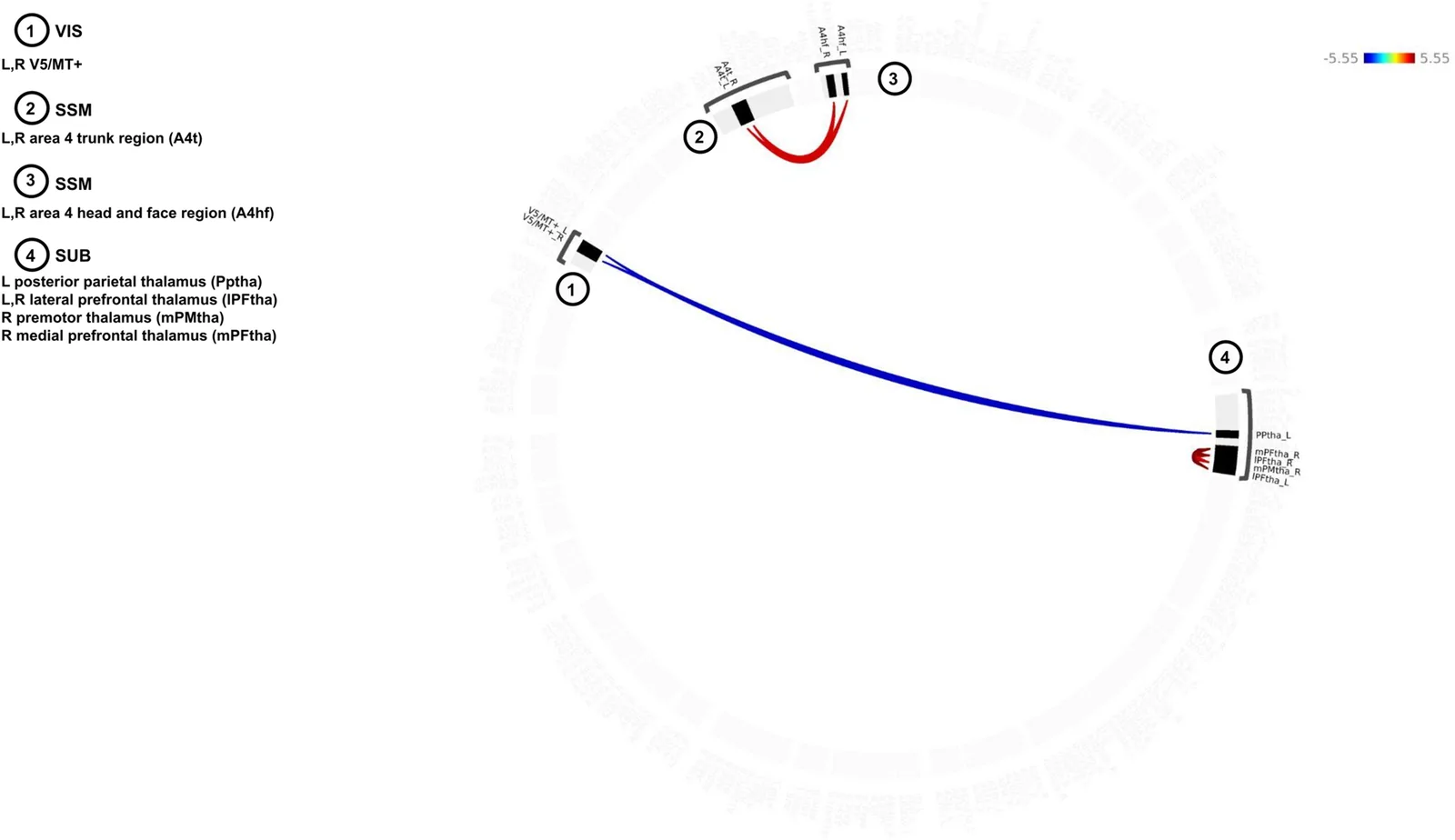
Thresholded functional connectivity map for the between-subject [CON (−1) vs. AVH+ (+1)] contrast for the *word-list* task (post-Bonferroni correction). Nodes are grouped circumferentially by functional network affiliation, showing the refined subset of 11 ROIs. Line thickness reflects *t*-statistic magni*t*ude.

Schizophrenia with auditory verbal hallucinations (AVH) is associated with sensorimotor and thalamocortical dysconnectivity that disrupts inner speech generation, sensory integration, and predictive monitoring. In the AVH+ (*N* = 23) vs. CON (*N* = 25) comparison during the word-list task, FNC revealed significant clusters across visual, sensorimotor, and subcortical networks. Within the visual network, bilateral V5/MT + , regions involved in motion perception and audiovisual integration, showed altered connectivity. Abnormal activity in these areas may reflect impaired predictive coding or excessive cross-modal binding, contributing to the externalization of hallucinated voices [34–38].

In the sensorimotor network, bilateral A4hf and A4t (subdivisions of primary motor cortex) were implicated. A4hf, responsible for articulatory control and inner speech regulation, may exhibit altered co-activation linked to involuntary speech generation or poor efference copy transmission. Subcortical findings included five thalamic ROIs (mPFtha, mPMtha, Pptha, and bilateral lPFtha), which connect to prefrontal and parietal hubs involved in reality monitoring, top-down executive control, and sensory filtering [34–38].

#### Activation maps and statistical energy profiles of paired states: AVH + vs. CON – Word list task.

Across the 11 FNC-derived ROIs, there were 2^11^ = 2,048 possible binary network states. After Bonferroni correction, four significant pairs of activation states were identified. In Fig 3, activation maps of paired states illustrate co-active and co-inactive ROI configurations.

**Fig 3:**
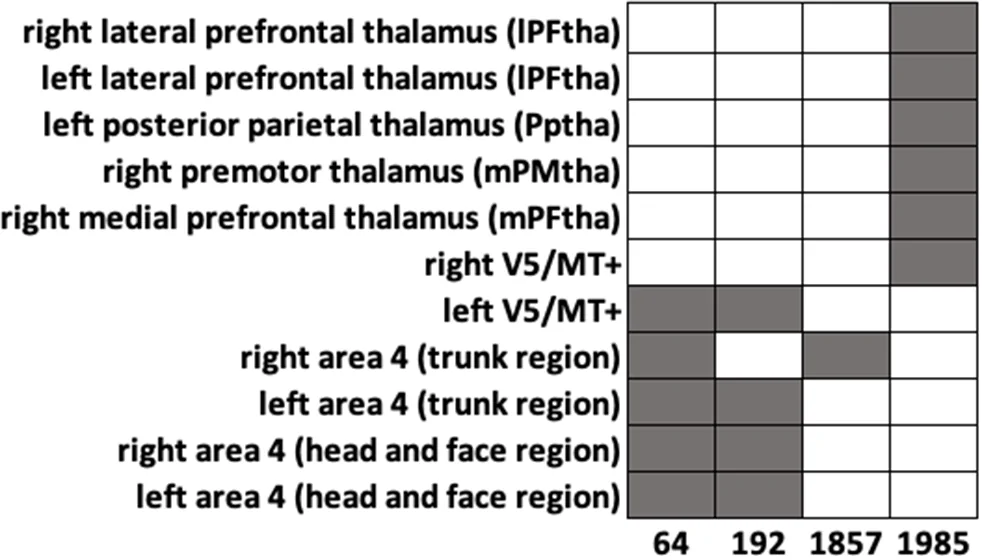
Binary state configurations across 11 FNC-derived ROIs (2^11^ = 2,048 possible states). Four state pairs (States 64, 192, 1857, and 1985) showed significant group differences after Bonferroni correction, with paired States 64 and 1985 serving as local energy minima (attractor basins). Active ROIs (+1) and inactive ROIs (−1) indicate synchronized co-activation patterns.

Energy distributions across these significant state pairs (States 64, 192, 1857, and 1985) are illustrated in Fig 4. The CON group demonstrated significantly lower statistical energy values across these attractor states compared to the AVH+ group (*p* < 0.05, Bonferroni-corrected). In the MEM statistical physics framework ($P(S)\propto e^{-E(S)}$), these lower energy values reflect higher state occupancy probabilities and deeper, more stable attractor basins in healthy controls, whereas elevated statistical energy in AVH+ indicates reduced state stability and increased network volatility during auditory processing [19,34,35].

**Fig 4:**
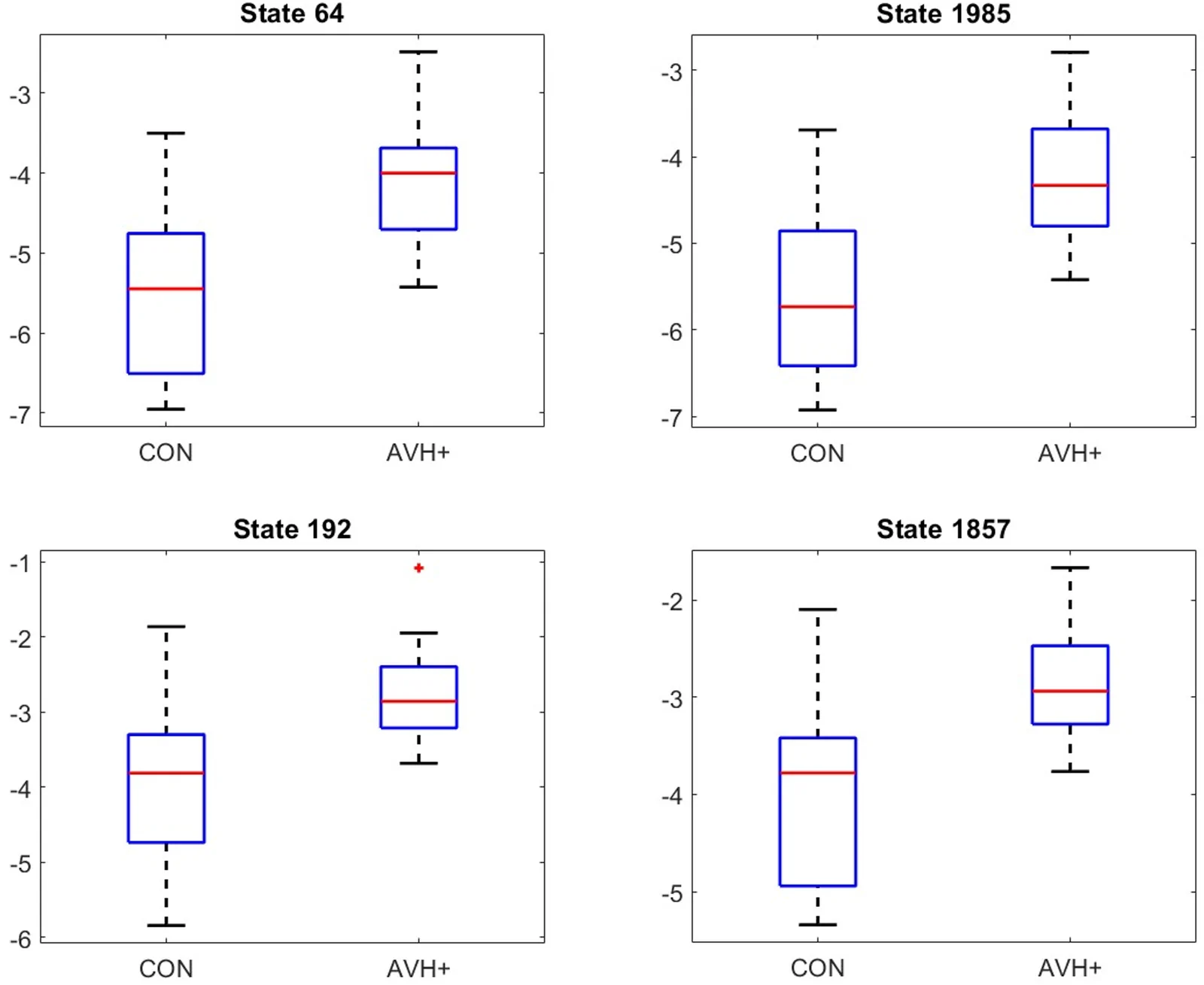
Boxplots comparing statistical energy *E*(*S*) between CON and AVH+ groups across significant state pairs (64 and 1985, 192 and 1857) for the *word-list* task. Lower statistical energy in the CON group reflects significantly deeper attractor basins and higher state occurrence probabilities ($P(S)\propto e^{-E(S)}$) compared to the AVH+ group.

### Spatial pairwise connectivity

#### Connectivity maps: AVH + vs. CON – Word list task.

We further validated these findings using Spatial Pairwise Connectivity (SPC) to capture localized thalamocortical interactions. Similar to the FNC pipeline, the unthresholded SPC connectome ring for the CON vs. AVH+ contrast exhibits a dense correlation network prior to correction (Fig 5). Following Bonferroni correction, the connectivity map was refined to display only significant ROI-to-ROI signatures (Fig 6), with nodes organized circumferentially by functional network affiliation and weak edges (*r* < 0.30) filtered to highlight core driving connections (SI Section S5).

**Fig 5:**
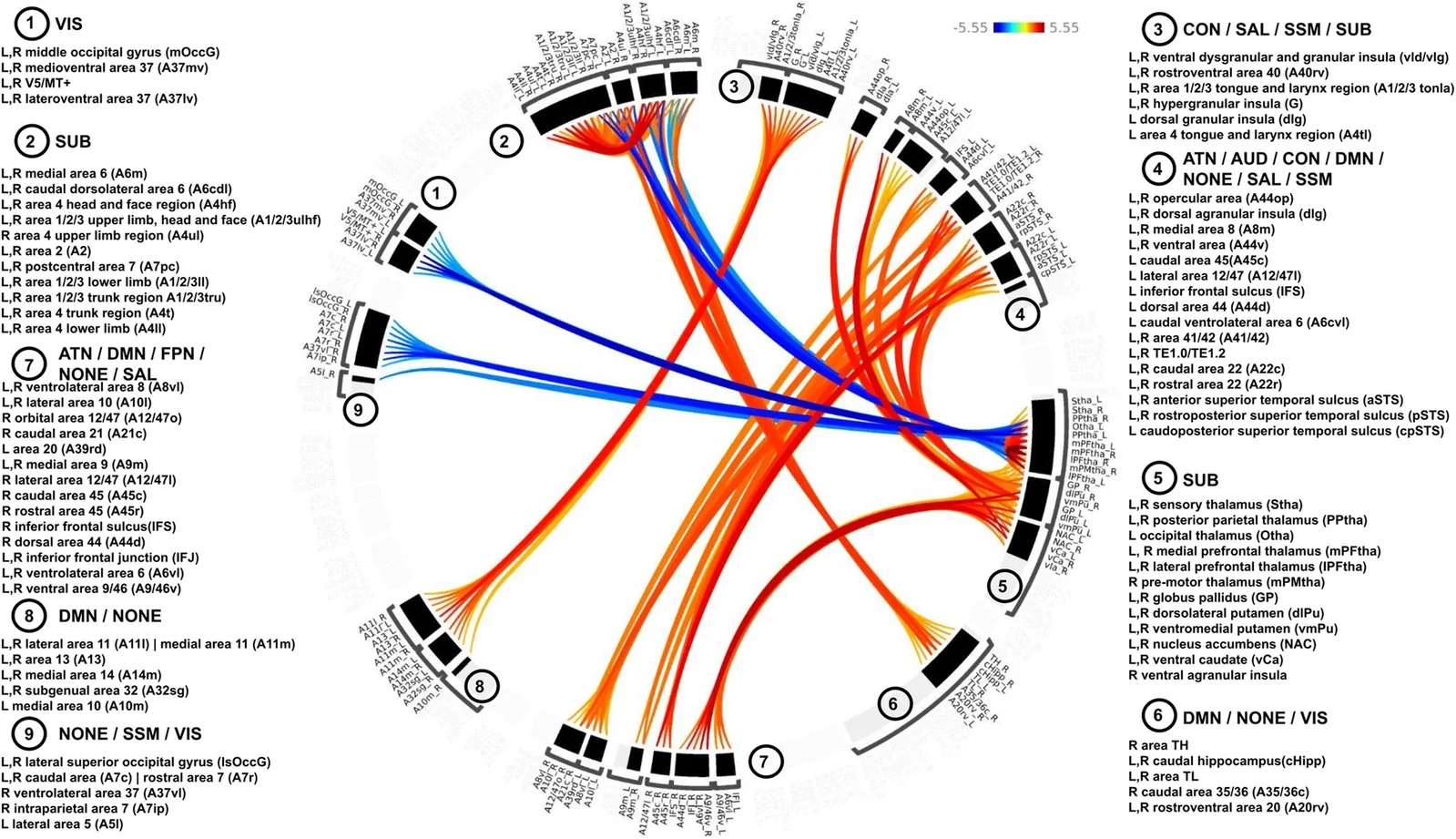
Unthresholded mean functional connectivity for the between-subject [CON (−1) vs. AVH+ (+1)] contrast presented as a connectome ring for the *word-list* task (before Bonferroni correction) computed using Spatial Pairwise Connectivity (SPC). CON: healthy controls; AVH + : patients with auditory verbal hallucinations.

**Fig 6:**
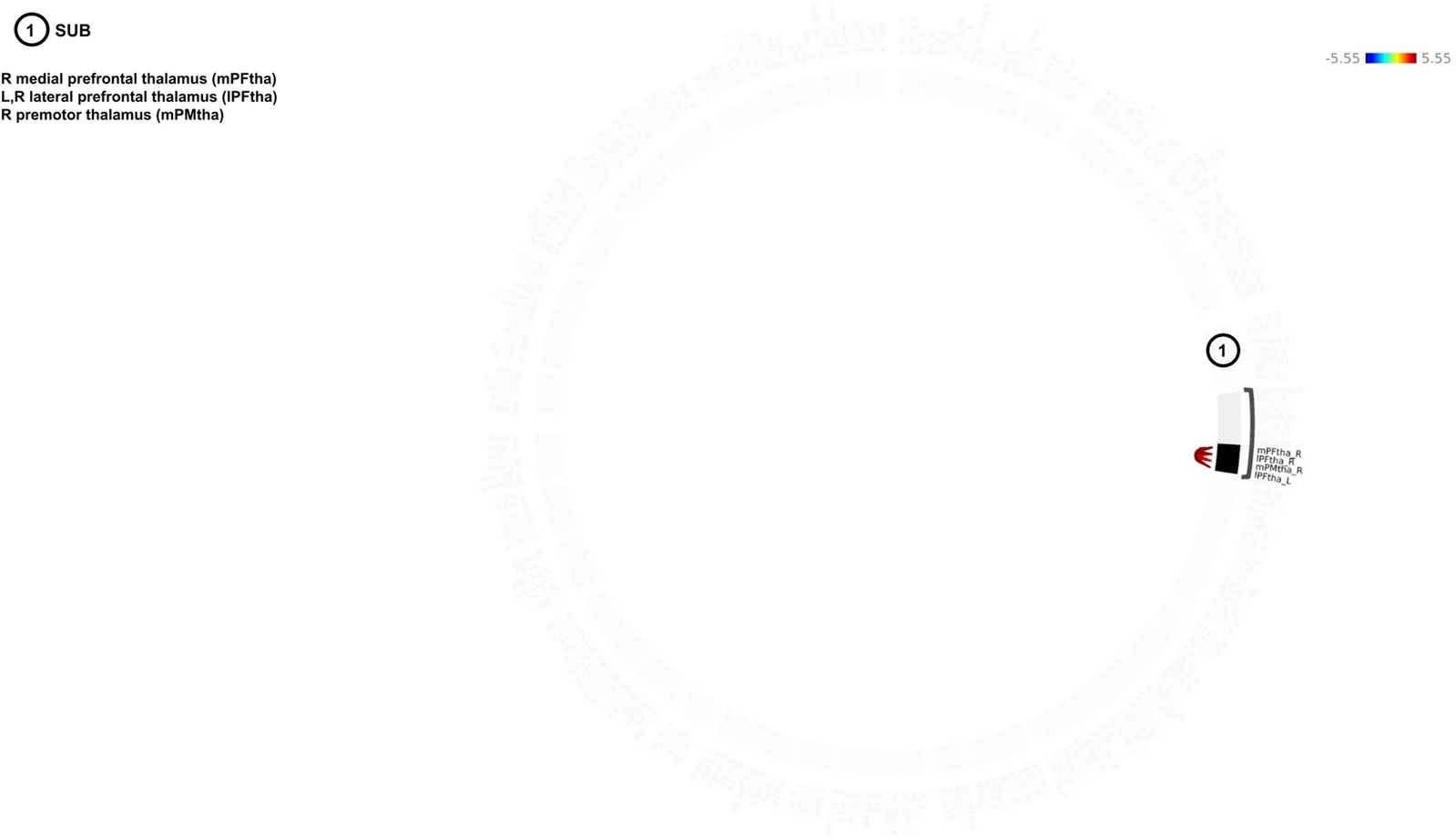
Thresholded functional connectivity map for the between-subject [CON (−1) vs. AVH+ (+1)] contrast for the *word-list* task (post-Bonferroni correction) computed using Spatial Pairwise Connectivity (SPC). Nodes are grouped circumferentially by functional network affiliation, displaying the refined subset of 4 thalamic ROIs.

Schizophrenia with AVH is associated with thalamocortical dysconnectivity affecting sensory gating, working memory, and top-down executive control. In the AVH+ (*N* = 23) vs. CON (*N* = 25) comparison during the word-list task, SPC identified four significant subcortical ROIs: right medial prefrontal-thalamus (mPFtha), right premotor-thalamus (mPMtha), and bilateral lateral prefrontal-thalamus (lPFtha).

The mPFtha, connected to the medial prefrontal cortex (mPFC), supports executive monitoring and reality monitoring. Dysconnectivity in this circuit is associated with altered top-down regulation, predisposing patients to the misattribution of internal thoughts as external voices. The mPMtha, linked to premotor and supplementary motor areas, contributes to motor planning and inner speech regulation. Dysfunction in this pathway is linked to disrupted inner speech monitoring, potentially resulting in unmonitored internal speech generation without adequate efference copy transmission. Finally, bilateral lPFtha, connected to the dorsolateral prefrontal cortex (DLPFC), supports working memory and cognitive inhibition. Dysconnectivity in this pathway is associated with impaired suppression of intrusive internal speech, compromising the patient’s capacity to differentiate self-generated mental events from external acoustic stimuli [34–38].

#### Activation maps and statistical energy profiles of paired states: AVH + vs. CON (Word list task).

Binarization across the four SPC-derived thalamic ROIs yielded 2^4^ = 16 possible binary network state configurations. Following Bonferroni correction, States 1 and 16 emerged as significant paired local energy minima (attractor basins) for both CON and AVH+ groups. Activation maps for these paired states illustrate synchronized co-activation (+1) and co-inactivation (−1) patterns across subcortical hubs (Fig 7).

**Fig 7:**
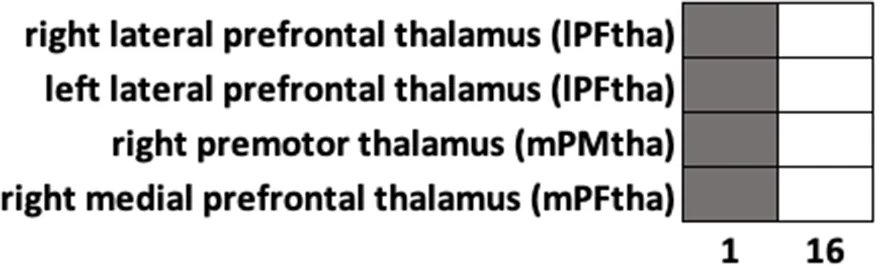
Binary state configurations across 4 SPC-derived thalamic ROIs (2^4^ = 16 possible states). States 1 and 16 were identified as significant local minima (attractor basins) following Bonferroni correction. Activation maps indicate synchronized co-active (+1) and co-inactive (−1) ROI configurations.

Boxplots comparing the statistical energy *E*(*S*) of these two attractor states are shown in Fig 8. The CON group exhibited significantly lower statistical energy values in States 1 and 16 relative to the AVH+ group (*p* < 0.05, Bonferroni-corrected). In accordance with the Maximum Entropy Model formulation ($P(S)\propto e^{-E(S)}$), lower statistical energy in healthy controls corresponds to deeper attractor basins and higher state occupancy probabilities, whereas elevated energy in AVH+ indicates compromised state stability across thalamocortical circuits during word-list processing.

**Fig 8:**
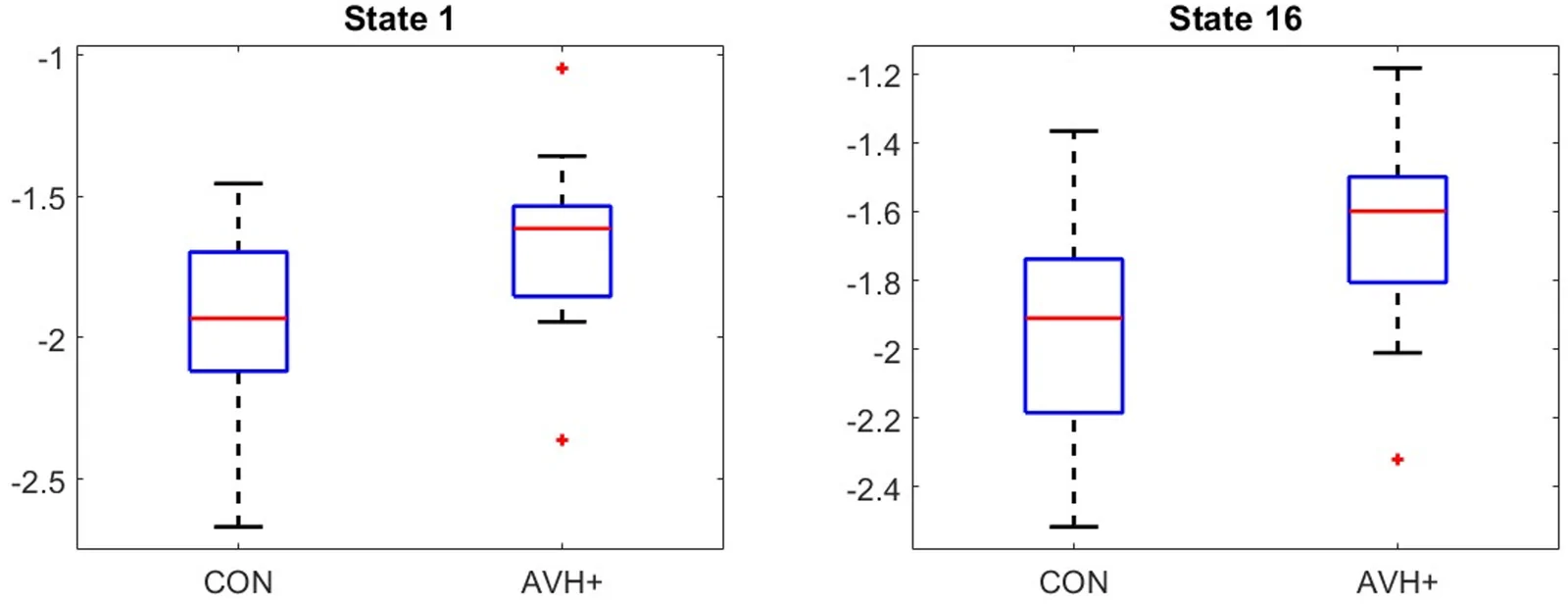
Boxplots comparing statistical energy *E*(*S*) between CON and AVH+ groups across significant SPC attractor states (States 1 and 16) for the *word-list* task. Results are derived from the CONN toolbox and energy landscape modeling. Lower statistical energy in healthy controls reflects significantly deeper attractor basins and higher state occurrence probabilities ($P(S)\propto e^{-E(S)}$) relative to the AVH+ group.

### State transition dynamics

State transition matrices derived from functional connectivity (FNC) and spatial pairwise connectivity (SPC) analyses revealed distinct patterns during the word list task in CON and AVH+ patients.

For FNC, of the Bonferroni-significant states 64, 192, 1857, and 1985—two states (64 and 1985) were identified as local minima. These states showed higher self-transition probabilities for CON (Table 5) than AVH+ patients as shown in Table 6. CON had longer dwell times than AVH+ patients (Table 7) and more visit counts (Table 8). These states appear to represent stable, low-energy configurations associated with efficient neural processing. In contrast, AVH+ patients exhibited more frequent transitions and shorter dwell times, suggesting less stable network dynamics and elevated energy demands.

**Table 5:** State transition matrix for the CON group using functional network connectivity (FNC), for the *word lists* task.

	State 64	State 192	State 1857	State 1985
**State 64**	0.35	0.06	0.00	0.00
**State 192**	0.14	0.14	0.00	0.00
**State 1857**	0.00	0.00	0.17	0.17
**State 1985**	0.00	0.00	0.03	0.41

**Table 6:** State transition matrix for the AVH+ group using functional network connectivity (FNC), for the *word lists* task.

	State 64	State 192	State 1857	State 1985
**State 64**	0.24	0.04	0.00	0.00
**State 192**	0.19	0.06	0.00	0.00
**State 1857**	0.00	0.00	0.11	0.05
**State 1985**	0.00	0.00	0.01	0.26

**Table 7:** Comparison of average dwell time for CON and AVH+.

State	Avg. Dwell Time (CON)	Avg. Dwell Time (AVH+)
**64**	1.49	1.19
**192**	1.02	0.49
**1857**	0.90	0.74
**1985**	1.55	1.28

**Table 8:** Comparison of total visit counts for CON and AVH+.

State	Total Visit Count (CON)	Total Visit Count (AVH+)
**64**	253	82
**192**	59	16
**1857**	48	19
**1985**	301	82

SPC analysis similarly identified states 1 and 16 as local minima, with CON again showing higher self-transition probabilities (Table 9) than AVH+ patients (Table 10). CON also had longer dwell times (Table 11) and more visit counts than AVH+ (Table 12). These patterns reinforce the idea that healthy brains tend to occupy energetically favorable (i.e., highly probable statistical attractor states) and functionally efficient states for longer durations, supporting sustained attention, working memory, and reality monitoring.

**Table 9:** State transition matrix for the CON group using spatial pairwise connectivity (SPC), for the *word lists task.*

	State 1	State 16
**State 1**	0.64	0.03
**State 16**	0.03	0.64

**Table 10:** State transition matrix for the AVH+ group using spatial pairwise connectivity (SPC), for the *word lists* task.

	State 1	State 16
**State 1**	0.59	0.03
**State 16**	0.02	0.59

**Table 11:** Comparison of average dwell time for CON and AVH+.

State	Avg. Dwell Time (CON)	Avg. Dwell Time (AVH+)
**1**	2.81	2.44
**16**	2.76	2.45

**Table 12:** Comparison of total visit counts for CON and AVH+.

State	Total Visit Count (CON)	Total Visit Count (AVH+)
**1**	2156	1697
**16**	2163	1746

Physiologically, lower energy states may reflect more statistically stable and computationally efficient functional network configurations, while longer dwell times suggest greater neural stability and resistance to perturbation. These dynamics are consistent with robust cognitive control and the suppression of intrusive verbal content—functions often impaired in schizophrenia.

The instability and elevated energy observed in AVH+ patients may reflect a breakdown in predictive coding, where top-down expectations override bottom-up sensory input, resulting in misattributed internal speech. Modeling these brain state transitions and energy landscapes could inform biologically grounded AI architectures capable of filtering internally generated noise and maintaining representational stability.

Together, these results highlight distinct physiological and computational differences in brain state stability and network efficiency between patients and CON. Future work could explore how these states relate to behavioral performance and whether they can be modulated to improve cognitive function or reduce hallucinatory symptoms.

### Correlation of brain energy with symptom severity

To evaluate the clinical relevance of the identified energy landscapes, we performed a post-hoc correlation analysis between the mean brain energy of Bonferroni-significant states and symptom severity (PSYRATS total score) within the AVH+ group (*N* = 23). Our analysis revealed a moderate positive correlation between energy in FNC State 192 and total PSYRATS scores (*r* = 0.32, *p* = 0.14), suggesting a trending relationship where higher energy—indicative of lower neural stability—is associated with more severe hallucinatory experiences. Correlations for other significant FNC and SPC states did not reach statistical significance (all *p* > 0.14), suggesting that while group-level differences in energy dynamics are robust, their direct linear relationship with symptom scales may be complex or limited by the sample size.

## Discussion

### FNC physiology: CON vs. AVH + – Word lists task

Functional network connectivity (FNC) analyses examined sensorimotor and thalamocortical dysconnectivity in schizophrenia patients with auditory verbal hallucinations (AVH), focusing on how disrupted brain networks contribute to inner speech generation, motor perception, and reality monitoring. Altered connectivity in these systems has been widely implicated in AVH pathophysiology, particularly through failures of predictive coding, corollary discharge, and self-monitoring mechanisms [11–13,39]. FNC analysis during the word list task revealed significant group differences between AVH+ patients (*N* = 23) and healthy controls (CON, *N* = 25), identifying key regions across visual, sensorimotor, and subcortical networks.

**Visual Network Dysfunction:** The left and right V5/MT+ regions—critical for motion processing and audiovisual integration—showed altered connectivity in AVH+ patients. Abnormal MT+ responses have been observed in schizophrenia and are associated with impaired motion perception and sensory integration [40]. MT+ interacts with V1, STS, parietal, and prefrontal regions, supporting predictive coding and multimodal binding [38]. Dysfunction here may reflect excessive audiovisual coupling or weakened sensory prediction, both of which can enhance the externalization of internally generated auditory content. Abnormal cross-modal integration has also been described in schizophrenia, further supporting this interpretation [41–44].

**Sensorimotor Network Dysconnectivity:** ROIs in the primary motor cortex (M1)—specifically the head/face (A4hf) and trunk (A4t) regions—were implicated in AVH+ patients. A4hf contributes to articulatory control and subvocal speech production. Previous work links abnormal motor cortex activation and involuntary subvocalization to AVH [45], consistent with theories of defective copy signaling [39]. Hyperactivity may amplify covert speech, while hypoactivity may weaken motor predictions, impairing the recognition of self-generated inner speech. A4t, involved in postural and bodily integration, may reflect broader embodied disturbances sometimes observed in schizophrenia.

**Subcortical Network Disruption:** Thalamic ROIs—including the right medial prefrontal–thalamus (mPFtha), right premotor–thalamus (mPMtha), left posterior parietal–thalamus (Pptha), and bilateral lateral prefrontal–thalamus (lPFtha)—support executive function, motor planning, and sensory integration. Extensive evidence shows that thalamocortical circuits are altered in schizophrenia [35–37]. Disruption in mPFtha and mPMtha may impair regulation of internal speech and cognitive control, while dysfunction in Pptha and lPFtha—connected to posterior parietal cortex and DLPFC—may weaken attentional gating and working memory functions [34,35]. Together, these abnormalities compromise the efference copy mechanism, leading to misattribution of internal speech as external [12,39].

**State-Based Biomarkers and Energy Dynamics:** Energy landscape analysis identified several Bonferroni-significant activation states (States 64, 192, 1857, and 1985) that differentiated AVH+ patients from CON. Similar statistical mechanics approaches have previously been shown to capture unstable attractor dynamics in psychiatric populations [14,15,19,31–33]. CON exhibited lower statistical energy (reflecting deeper attractor basins and higher state probability density) and longer dwell times, indicating more stable network configurations. Conversely, AVH+ patients showed higher statistical energy and reduced dwell times—patterns consistent with neural state volatility, shallower attractor basins, and impaired cognitive filtering.

## SPC physiology: CON vs. AVH + – Word lists task

Spatial pairwise connectivity (SPC) analyses further highlighted thalamocortical disruptions. Four subcortical ROIs—mPFtha, mPMtha, and bilateral lPFtha—showed significant differences between AVH+ patients and CON.

**Medial Prefrontal-Thalamus (mPFtha):** mPFtha connects with the medial prefrontal cortex, supporting executive function, salience processing, and reality monitoring. Thalamic–prefrontal dysconnectivity is a well-established feature of schizophrenia and is associated with cognitive control and self-monitoring deficits [34–37]. Altered mPFtha activity may thus impair the suppression of intrusive internal speech, contributing to AVH.

**Premotor-Thalamus (mPMtha):** This region supports motor planning and inner speech regulation. Abnormal premotor–thalamic signaling can disrupt the internal monitoring of self-generated speech, paralleling findings of reduced corollary discharge in AVH [39,45].

**Lateral Prefrontal-Thalamus (lPFtha):** Connected to the DLPFC, lPFtha mediates working memory and inhibitory control. Schizophrenia is characterized by reduced DLPFC engagement and attentional dysregulation [34,35]. Dysconnectivity in this pathway may impair the filtering of internally generated verbal content, reinforcing the externalization of inner speech.

**State-Based Biomarkers and Energy Dynamics:** SPC revealed four Bonferroni-significant activation states (States 1, 4, 13, and 16), with States 1 and 16 serving as local minima—potential biomarkers of network stability. CON exhibited lower statistical energy and longer dwell times, consistent with efficient, stable thalamic–prefrontal dynamics. In contrast, AVH+ patients showed elevated energy and unstable transitions, akin to attractor dynamics in computational systems when internal representations become noisy and unreliable [30–33].

**Interpretation and Integration:** Together, the FNC and SPC analyses converge on a key observation: AVH are significantly associated with disrupted thalamic–sensorimotor–prefrontal circuitry. These circuits are theorized to support predictive coding, efference copy, and reality monitoring—mechanisms that may be essential for distinguishing self-generated thoughts from external stimuli [11–13,38,39]. The instability observed in energy landscape dynamics is consistent with computational models of dysregulated attractor states in schizophrenia, where noisy internal predictions may produce perceptual distortions resembling hallucinations [14,15,19,31–33]. These findings highlight network-level potential biomarkers of AVH and underscore the promise of task-based connectivity and energy-based modeling for characterizing perceptual disturbances in schizophrenia.

To further evaluate clinical relevance, we investigated the relationship between neural stability and symptom severity. While the correlations between energy levels and total PSYRATS scores did not reach the threshold for statistical significance, FNC State 192 showed a moderate positive association (*r* = 0.32, *p* = 0.14). This trend is consistent with the hypothesis that higher neural energy—representing a more unstable attractor state—tracks with more intense clinical phenotypes. The lack of a strong linear correlation across all states may reflect the heterogeneity of the AVH phenotype or the constraints of using a single global symptom scale to capture complex dynamic transitions.

### Limitations and generalizability

While this study provides evidence for energy landscape differences in schizophrenia, certain limitations must be acknowledged. First, as a secondary analysis of a publicly available dataset, our choice of behavioral metrics was limited to those provided in the original study (e.g., IQ and PSYRATS). Future prospective studies should include a broader battery of real-time behavioral tasks to further validate the link between state stability and cognitive performance. Second, while our sample size is consistent with standard neuroimaging cohorts, the results may not be fully generalizable to all stages of the schizophrenia spectrum, particularly chronic populations or those under different medication regimens.

## Conclusion

This study provides converging evidence that auditory verbal hallucinations (AVH) in schizophrenia are linked to widespread dysconnectivity across visual, sensorimotor, and thalamocortical networks. Task-based fMRI revealed that AVH+ patients exhibit unstable brain state transitions, elevated statistical energy dynamics, and reduced dwell times—hallmarks of neural state instability and impaired cognitive filtering. Preliminary clinical correlations suggest that these energy landscapes may track with symptom severity, providing a potential bridge between macroscopic network dynamics and clinical presentation. Functional network connectivity (FNC) and spatial pairwise connectivity (SPC) analyses identified key disruptions in regions responsible for inner speech generation, motor feedback, and executive control, including V5/MT + , A4hf, mPFtha, mPMtha, and lPFtha.

These findings support a systems-level account of hallucinations as emergent phenomena potentially driven by dysregulated predictive coding and impaired reality monitoring. The observed disruption of the efference copy mechanism, combined with hyperactive sensory integration and weakened top-down inhibition, may contribute to a state where internally generated speech is misattributed as external auditory input. This breakdown in generative control mirrors attractor instability in artificial neural networks, where noisy internal representations can produce fluent but spurious outputs—akin to hallucinations in both biological and computational systems.

Future research is expected to delve deeper into the neural mechanisms underlying predictive coding and generative control, potentially identifying specific circuit-level codes that govern cognitive filtering and sensory integration. By mapping how neural information processing might resemble computational principles—such as those seen in advanced artificial systems—this line of inquiry may bridge gaps between neuroscience and machine learning.

Such interdisciplinary efforts hold promise not only for advancing our understanding of schizophrenia and other neuropsychiatric disorders but also for informing the development of novel biomarkers and targeted therapies. Ultimately, exploring these predictive architectures at both biological and computational levels could pave the way for innovative interventions and inspire new paradigms in artificial intelligence design, grounded in the complexities of brain function.

## Supporting information

S1 Appendix Supplementary procedural details, atlas mappings, and secondary task analyses. Contains detailed Maximum Entropy Model formulations, motion scrubbing protocols, secondary task comparisons, and Brainnetome ROI mappings.
(PDF)

S1 File Brainnetome Atlas subregion definitions and peak coordinates. Spreadsheet containing all 246 ROI labels, MNI coordinates, and macro-network assignments.
(XLSX)
