# Supplementary material for: Energy landscape and dynamic state modeling for schizophrenia biomarker extraction in task-based fMRI
Source: PLoS One. 2026 Oct 8;21(10):e0339756. doi: 10.1371/journal.pone.0339756 (PMC13649140; doi:10.1371/journal.pone.0339756)
Supplement: Supplementary file 1 [file pone.0339756.s001.pdf]

Supplementary Information (S1 Appendix): Energy Landscape and Dynamic State Modeling for Schizophrenia Biomarker Extraction in Task-Based fMRI

Contents

S1 Pipeline Reproducibility and Preprocessing Parameters **2**
SS1.1 Software Environment and Toolboxes 2
SS1.2 Preprocessing and Denoising Specifications 2
SS1.3 Quality Control and Participant Cohort 2

S2 Brainnetome Atlas ROI Reference and Network Assignments **3**
SS2.1 Primary Atlas Regional Mapping 3

S3 Task Selection Rationale and Comparative Analyses **3**
SS3.1 Rationale for Primary Word-List Paradigm Focus 3

S4 Exploratory Energy Landscape Analyses across Secondary Tasks **3**
SS4.1 FNC-Derived ROI Subsets 4
SS4.2 SPC-Derived ROI Subsets 4

S5 Maximum Entropy Model (MEM) and Statistical Energy Formulation **4**
SS5.1 Binarization Protocol 4
SS5.2 Pairwise Maximum Entropy Model Fitting 4
SS5.3 Conceptual Distinction: Statistical Energy versus Metabolic Energy 5

S6 High-Resolution Connectome Visualizations and Visual Conventions **5**

Overview and Code Availability

This Supplementary Information document provides full procedural details, mathematical formulations, software parameters, and secondary task analyses supporting the main manuscript. To ensure complete computational reproducibility, all custom scripts used for functional MRI preprocessing, Region of Interest (ROI) time-series extraction, Maximum Entropy Model (MEM) fitting, energy landscape generation, and connectome visualization have been archived and made publicly available.

The complete codebase (`energy-landscape-analysis-master`), processed ROI matrices, Brainnetome atlas sub-region files (`BrainnetomeAtlas_subregions_finalized.xlsx`), and script execution pipelines are available in our public GitHub repository at `https://github.com/janerradallen/sz-task-based/tree/main`.

S1 Pipeline Reproducibility and Preprocessing Parameters

SS1.1 Software Environment and Toolboxes

Functional MRI data were processed using MATLAB (version R2023b; MathWorks, Natick, MA) alongside SPM12 (Wellcome Department of Cognitive Neurology, London, UK) and the CONN Functional Connectivity Toolbox (version 22.a; `https://www.nitrc.org/projects/conn`). Time-series extraction from specific atlas regions was executed using the REX toolbox. Downstream Maximum Entropy Modeling and energy landscape numerical optimizations were conducted using custom MATLAB functions located within the repository directory `energy-landscape-analysis-master/`.

SS1.2 Preprocessing and Denoising Specifications

Functional images underwent standard spatial preprocessing within the CONN environment. Slice-timing correction was applied to adjust for acquisition time differences across slices. Reorientation and spatial realignment were performed to estimate six rigid-body head motion parameters. Functional images were co-registered to each participant's high-resolution T1-weighted anatomical scan, normalized to MNI152 space, and spatially smoothed using an isotropic Gaussian kernel with a full width at half maximum (FWHM) of 6 mm.

Denoising was performed using Ordinary Least Squares (OLS) linear regression within CONN's anatomical component-based noise correction framework (aCompCor; Behzadi et al., 2007). Potential confounding effects regressed out from each voxel's BOLD time series included:

- Principal noise components extracted from eroded white matter (5 components) and cerebrospinal fluid (5 components) masks (Chai et al., 2012);
- Twelve head motion parameters (3 translational and 3 rotational parameters, plus their first-order temporal derivatives; Friston et al., 1995);
- Scrubbing regressors consisting of individual indicator (dummy) variables for each identified outlier volume (FD $>$ 0.5 mm or global signal changes $>$ 3 SD; Power et al., 2014) to eliminate artifactual variance;
- Main task effects convolved with the canonical hemodynamic response function (HRF; Whitfield-Gabrieli & Nieto-Castanon, 2012) to isolate task-driven fluctuations from intrinsic background signal.

A temporal bandpass filter of 0.01–0.1 Hz was applied to reduce low-frequency drift and high-frequency physiological noise.

SS1.3 Quality Control and Participant Cohort

Head motion was quantitatively compared across groups following denoising. No statistically significant differences in mean Framewise Displacement. The analyzed cohort comprised $N = 71$ participants (CON = 25, AVH+ = 23, AVH- = 23). Sex and smoking status were included as design covariates across all group comparisons.

S2 Brainnetome Atlas ROI Reference and Network Assignments

Primary ROI-to-ROI connectivity matrices were derived using the 246 subregions defined by the human Brainnetome Atlas (BNA-246). These subregions were assigned to major canonical intrinsic connectivity networks, including the Auditory Network (AUD), Attention Network (ATN), Cingulo-Opercular Network (CON), Default-Mode Network (DMN), Frontoparietal Network (FPN), Salience Network (SAN), Sensorimotor Network (SSM), Subcortical Network (SUB), and Visual Network (VIS). Full regional definitions, MNI centroid coordinates, and subnetwork assignments are archived in the project repository file `BrainnetomeAtlas_subregions_finalized.xlsx`.

SS2.1 Primary Atlas Regional Mapping

Table S1 lists the indices, anatomical labels, MNI peak coordinates, and network affiliations for the subset of ROIs identified via Spatial Pairwise Connectivity (SPC) clustering that demonstrated significant group differences following Bonferroni correction ($\alpha = 0.05/\text{total clusters}$).

Table S1: **Brainnetome Atlas Subregions: Superior Frontal Gyrus Definitions and Network Affiliations.**

Lobe	Gyrus	ID (L)	ID (R)	Anatomical / Cytoarchitectonic	Subnetwork
Frontal Lobe	SFG, Superior Frontal Gyrus	1	2	A8m, medial area 8	CON
		3	4	A8dl, dorsolateral area 8	DMN
		5	6	A9l, lateral area 9	DMN
		7	8	A6dl, dorsolateral area 6	CON
		9	10	A6m, medial area 6	Sensorimotor
		11	12	A9m, medial area 9	DMN
		13	14	A10m, medial area 10	DMN

Note: Full regional names, MNI centroid coordinates, and network labels are archived in the repository dataset file: `https://github.com/janerradallen/sz-task-based/blob/main/BrainnetomeAtlas_subregions_finalized.xlsx`.

S3 Task Selection Rationale and Comparative Analyses

SS3.1 Rationale for Primary Word-List Paradigm Focus

Although functional data were acquired across three distinct auditory conditions (word lists, sentence lists, and reverse speech), the primary downstream Energy Landscape Modeling was focused on the word-list paradigm. This focus was established a priori based on the theoretical requirement that the task heavily engages language processing and auditory monitoring networks without confounding narrative synthesis. The word-list paradigm consistently engaged primary auditory cortex, superior temporal gyrus, and inferior frontal regions, offering maximum sensitivity to localized network dysregulation in AVH.

To ensure transparency, functional connectivity and GLM contrasts were evaluated across the sentence-list and reverse-speech tasks. Downstream energy landscape modeling conducted on these secondary conditions yielded qualitatively similar topological structures, but demonstrated weaker statistical group separation between AVH+ and AVH− cohorts compared to the word-list task. Consequently, downstream state-space modeling applied to secondary tasks is explicitly designated as exploratory and hypothesis-generating.

S4 Exploratory Energy Landscape Analyses across Secondary Tasks

To complement the primary *a priori* word-list analysis, energy landscape (EL) modeling was systematically evaluated across secondary auditory tasks and contrast conditions.

SS4.1 FNC-Derived ROI Subsets

Exploratory state-space analyses were conducted across the following Functional Network Connectivity (FNC) ROI clusters:

1. Controls vs. AVH+ during the sentence task ($N = 9$ ROIs)
2. Controls vs. AVH+ during the word task ($N = 9$ ROIs)
3. AVH+ vs. AVH− during the reverse speech task ($N = 5$ ROIs)
4. AVH+ vs. AVH− during the sentence task ($N = 7$ ROIs)
5. AVH+ vs. AVH− during the white noise condition ($N = 2$ ROIs)

SS4.2 SPC-Derived ROI Subsets

Exploratory state-space analyses were conducted across the following Spatial Pairwise Connectivity (SPC) ROI clusters:

1. Controls vs. AVH+ during the word task ($N = 4$ ROIs)
2. AVH+ vs. AVH− during the sentence task ($N = 6$ ROIs)
3. AVH+ vs. AVH− during the word task ($N = 6$ ROIs)

As noted in the main text, while secondary conditions revealed qualitative similarities in energy landscape architecture, the word-list task yielded the most robust statistical group differentiation between AVH+ and AVH− cohorts.

S5 Maximum Entropy Model (MEM) and Statistical Energy Formulation

SS5.1 Binarization Protocol

To construct the statistical state space, continuous BOLD time series extracted via the REX toolbox were binarized. For each participant, ROI time series were z-score normalized. The binarized state $s_i(t) \in \{-1, +1\}$ of region i at time point t was defined as:

$$s_i(t) = \begin{cases} +1, & \text{if } z_i(t) \geq 0 \\ -1, & \text{if } z_i(t) < 0 \end{cases} \tag{S1}$$

A global brain state at time t is represented by the binary vector $S(t) = [s_1(t), s_2(t), \ldots, s_N(t)]^\top$, yielding a total state-space size of 2^N possible configurations.

SS5.2 Pairwise Maximum Entropy Model Fitting

Following the principle of maximum entropy, the probability distribution $P(S)$ over all possible network states S was constrained to match the empirical activation rate of individual regions and the pairwise co-activation rates between regions. The resulting Boltzmann distribution is expressed as:

$$P(S) = \frac{\exp(-E(S))}{\sum_{\{S'\}} \exp(-E(S'))} \tag{S2}$$

where $E(S)$ represents the statistical energy of state S. The statistical energy function is explicitly defined as:

$$E(S) = -\sum_{i=1}^{N} h_i s_i - \frac{1}{2} \sum_{i=1}^{N} \sum_{j=1}^{N} J_{ij} s_i s_j \tag{S3}$$

In this formulation, h_i represents the intrinsic activation threshold (bias) of region i, and J_{ij} denotes the pairwise coupling parameter between regions i and j. Parameters h_i and J_{ij} were iteratively estimated using gradient descent optimization until the model-predicted first- and second-order moments converged with empirical observations (Pearson correlation $r > 0.98$).

SS5.3 Conceptual Distinction: Statistical Energy versus Metabolic Energy

It is critical to distinguish the term *statistical energy* $E(S)$ in Maximum Entropy Modeling from biological or metabolic energy expenditure (e.g., ATP or glucose consumption). In statistical physics, $E(S)$ is strictly a logarithmic transform of state probability ($E(S) \propto -\ln P(S)$).

A lower statistical energy value denotes a state with a higher occurrence probability within the system's state space, corresponding to a statistical *basin of attraction* or stable network configuration. Conversely, high energy states correspond to improbable network configurations. Unless combined with direct metabolic measurements (e.g., PET or arterial spin labeling), lower energy states in this framework signify statistical network stability, not reduced physiological energy demand.

S6 High-Resolution Connectome Visualizations and Visual Conventions

To ensure visual clarity and consistency across the functional network connectivity diagrams (Figures 1, 2, 5, and 6 in the main manuscript), the following visual conventions and thresholding parameters were applied:

1. **ROI Clustering and Spatial Organization:** Regions of interest (ROIs) along the connectome ring and matrix diagrams are hierarchically clustered based on functional network connectivity (FNC) and spatial pairwise connectivity (SPC) profile similarities as derived in the CONN environment, rather than fixed circumferential network ordering.

2. **Edge Thresholding:** Functional connections were thresholded using strict Bonferroni correction for multiple comparisons across ROI-to-ROI pairs to isolate statistically significant connections and focus visualizations on key differential pathways across groups.

3. **Color and Line Weight Conventions:** Line thickness (edge weight) scales proportionally with the absolute magnitude of functional connectivity (t-statistic strength). Red edges denote hyper-connectivity (increased functional coupling in AVH+ relative to controls or AVH−), whereas blue edges denote hypo-connectivity (decreased functional coupling).

Note on Network Mapping vs. Graphical Layout: While ROI nodes were assigned to 9 canonical functional networks (AUD, ATN, CON, DMN, FPN, SAN, SSM, SUB, VIS) mapping Brainnetome subregions based on AAL-inspired taxonomy for hypothesis testing, connectome circular plots display ROIs ordered via CONN's data-driven hierarchical clustering (FNC/SPC similarity). This visual layout clusters functional modules based on shared connectivity profiles to minimize cross-ring visual clutter without altering the underlying statistical network classifications.
